# Improving health equity among the African ethnic minority through health system strengthening: a narrative review of the New Zealand healthcare system

**DOI:** 10.1186/s12939-020-1125-9

**Published:** 2020-02-06

**Authors:** Blessing Kanengoni, Sari Andajani-Sutjahjo, Eleanor Holroyd

**Affiliations:** 1 School of Public Health and Psychosocial Studies, Auckland University of Technology, 90 Akoranga Drive, Northcote, Auckland, 0627 New Zealand; 2 Nursing Research, Department of Nursing, Auckland University of Technology, Auckland, New Zealand

**Keywords:** Health equity, Africans, Health delivery systems, Ethnic minority, New Zealand, Health system strengthening

## Abstract

**Background:**

In New Zealand, health equity is a pressing concern and reaching disadvantaged populations has become the goal to close the inequity gap. Building and strengthening health systems is one way to secure better outcomes. However, the discourse to date has predominately focussed on inequities in health outcomes for Māori. This study has interest in the African ethnic minority community in New Zealand. It undertakes a narrative review of the New Zealand health system which aims to identify literature around the attainment of health equity of African minority by: (i) providing a critical overview of the healthcare delivery system using World Health Organization’s six inter-related building blocks of health system strengthening; (ii) developing a summary and discussions of the research results and; (iii) identifying priorities and recommendations for future research.

**Method:**

A narrative review of 27 articles published between January 2010 and June 2019 were selected from CINAHL, PubMed, Scopus, Google Scholar. Grey literature also informed the review. Articles excluded studies: (i) non-related to New Zealand; (ii) with no focus on equity on ethnic minority in the delivering of healthcare; (iii) had no full text available.

**Findings:**

Literature on Africans health outcomes were scarce regarding the six building blocks. However, findings show inequities in accessibility of health services, a non-ethnic inclusive health workforce, a leadership and governance which lack political will on migrant health and resultantly an under-performing health information system which influences resource allocation.

**Recommendation and conclusion:**

An improvement and well-functioning health information system is pivotal to capture the unmet needs of the African population. There is a need for research and political will to invest in African minority health and diverse workforce that understands the background of the African population; and action to address structural and institutional racism and white privilege to address root causes of inadequate access and care processes for ethnic minorities.

## Introduction

As the growth of migrant communities continues to raise globally, creating unique health challenges at a scale that has never been seen, there is renewed interest in and momentum for strengthening health systems [1, 2]. There is mounting evidence that health systems that deliver services equitably and efficiently achieve health for all [3]. Health system strengthening is therefore both critical and timely as it comes in the wake of the 2030 Agenda for Sustainable Development call to “*leave no one behind”,* where State governments are encouraged to integrate the health needs of migrants into national plans, policies, and strategies across sectors [4]. The G20 also recently reaffirmed the importance of continuing to strengthen global health systems as health systems have neither been responsive enough to the migrant influx, nor to the moral requirement to attend to migrants with dignity [5]. Table 1 pre- 79 T1 sents the aims and desirable attributes of each building 80 block.

In light of above, there is growing recognition that the health of New Zealand citizens is inextricably related to the health of the estimated 1.2 million migrants residing in a community of 4.4 million [6]. Economic, human rights, and public health imperatives all point to the need to ensure that migrant populations have access to equitable, efficient and quality health services critical for achieving improved health status [7, 8], in order to have an impact on the health outcome for the entire community [3]. The Migration Integration Policy Index – a government sourced document report the New Zealand government to have taken official measures to build responsive health systems which are culturally competent and committed to properly treating patients of diverse cultural backgrounds by use of the Pacific and Māori health models which have been adapted to serve immigrant communities [9]. However, independent research in New Zealand show inequities remain high [10, 11]. There are substantial challenges to attaining health equity for migrants, in particular ethnic minority groups. The African com- 78 munity is one such migrant minority group.

These ethnic minorities often have reduced entitlements in receiving societies. Not only do they have reduced ac- 117 cess to health care for a number of political, administrative 118 and cultural reasons which are not necessarily present for 119 the native population [10, 24], they are exposed to poor 120 working and living conditions and or resource-poor 121 neighbourhoods [6, 25–27], which leads to poor health 122 outcomes. New Zealand comprises of many ethnic groups with the dominant groups being European ethnic group which makes up the majority of the population (74%), with Māori - *the Tangata Whenua -* of Indigenous people, being the largest minority (15%), followed by Asian (12%) and Pacific (7%) [12]. Resultantly, the current trends in health equity studies have been heavily focused on Māori [13–15], the Pacific [16] and to a lesser extent the Asian community [17, 18]. The Treaty of Waitangi of Biculturalism and the principles of protection, participation and partnership of the Māori indigenous of New Zealand, has therefore widely informed the health policies priorities and services in New Zealand [19]. The migrants from the Western Pacific regions came to New Zealand under the special arrangements between Pacific governments after attaining colonial independence; temporary seasonal work schemes and the family reunion and international/humanitarian streams in immigration policy; and more recently, the New Zealand’s special quota systems for Samoa and the New Zealand’s ‘Pacific Access Category’, which awards entree to a set number of migrants from Tonga, Kiribati and Tuvalu each year [20]. Therefore, the New Zealand health policies and services are informed by historical migration and within the protection of its indigenous Māori minorities. For that reason, it is of no surprise, that resources dedicated for migrants coming later in the early 1990s like African communities [21] have been lacking. The health and well-being of ethnic minority populations has therefore been historically and consistently underrepresented in New Zealand, yet New Zealand is a culturally diverse nation, which requires a set refugee and migrant health policies and strategies that respond to the health needs of migrants.

**Table 1:** The WHO health system framework

Building block	Aims and desirable attributes
Service delivery	To deliver effective, safe, quality personal and non-personal health interventions to those who need them, when and where needed, with minimum waste of resources.
Health workforce	A health workforce which works in ways that are responsive, fair and efficient to achieve the best health outcomes possible, given available resources and circumstances; i.e. there are sufficient numbers and mix of staff, fairly distributed; they are competent, responsive and productive.
Health information system	A health information system that ensures the production, analysis, dissemination and use of reliable and timely information on health determinants, health systems performance and health status.
Medical products/ vaccines/technologies	A health system that ensures equitable access to essential medical products, vaccines and technologies of assured quality, safety, efficacy and cost-effectiveness, and their scientifically sound and cost-effective use.
Health financing system	A health financing system that raises adequate funds for health, in ways that ensure people can use needed services, and are protected from financial catastrophe or impoverishment associated with having to pay for them.
Leadership and governance	To ensure strategic policy frameworks exist and are combined with effective oversight, coalition building, the provision of appropriate regulations and incentives, attention to system-design, and accountability.

*Source*: [3]

This article adopts the definition of ethnic minority to be an immigrant or racial groups or immediate descendant thereof [22], regarded by those claiming to speak for the cultural majority as distinct and unable to adapt and adjust with ease into the New Zealand society due to differences in cultures and traditions [23]. The definition is ‘migrant oriented’ and aligns well with this article as ethnic minority grouping generally becomes noticeable due to migration, whether forced or voluntary and may only attain the position of an ethnic association as a result of migration [22, 23]. This paper set out to undertake a situational analysis of the New Zealand health system in improving health and health equity amongst African ethnic minority using the World Health Organisation (WHO) framework on health system strengthening.

WHO developed two of these frameworks. The first framework recognised fundamental goals of the health systems (health status, financial risk protection, and responsiveness) and four functions of health systems that determine how inputs affect health systems performance (resource generation, financing, service provision, and stewardship) [28]. In this framework, Musgrove and colleagues [28], defined health systems as including all actors, institutions, and resources whose primary intention were to promote, maintain, or restore health. The other WHO framework [3] built upon Musgrove’s framework by organizing health systems into six building blocks: service delivery, health workforce, information, medical products and technologies, financing, and governance and leadership. Since its launch in 2007, this framework has been used worldwide as it has been seen as the most comprehensive and it provides specific guiding blocks strengthening health system in many countries [3, 29]. This article, therefore adopted it to assess the New Zealand health system capacity to manage ethnic minorities with specific reference to the African community.

In this article, gaps and key constraints are critically reviewed for each building block where literature exists. Subsequently, recommendations are provided to government, stakeholders and health and social services professionals, which enables the management and the collaborations of the building blocks in ways that ensure the equitable and sustained improvement of health services and health outcomes for the African population.

## Method

**Fig. 1:**
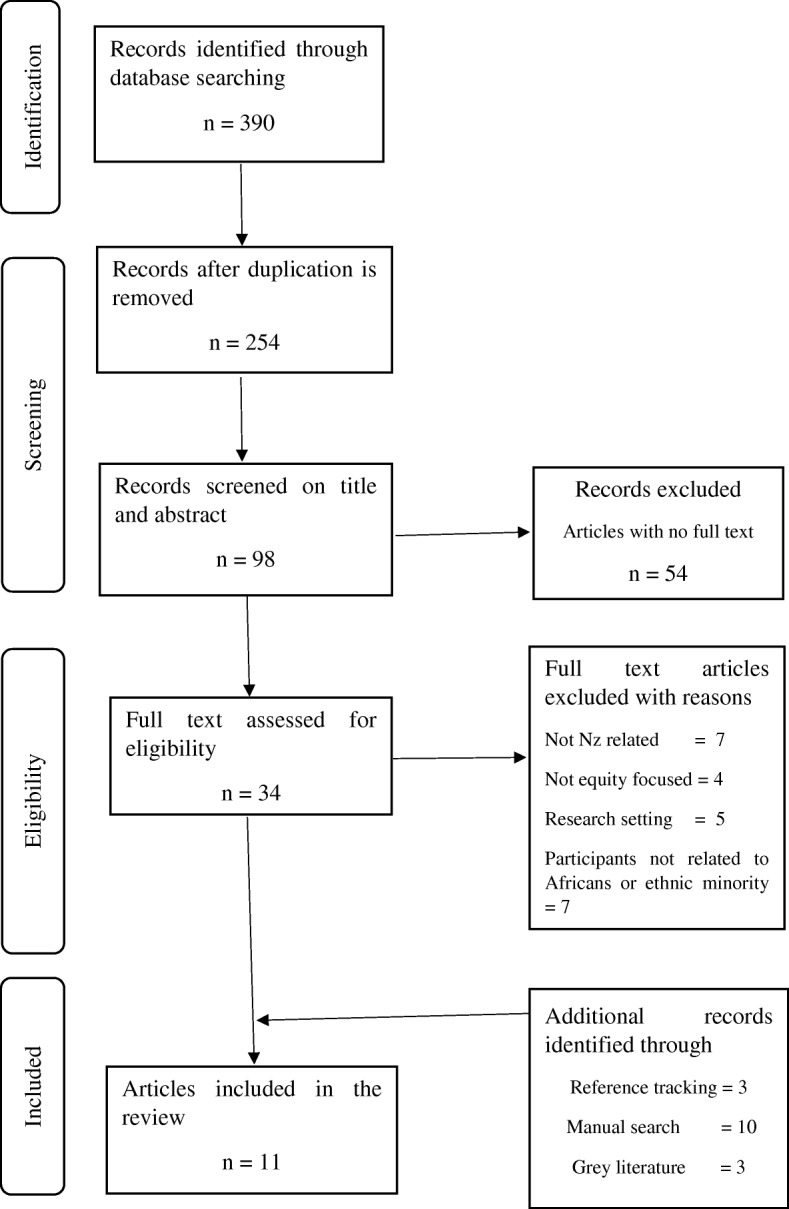
Preffered Reporting Items for Systematic Reviews and Meta-Analyses (PRISMA) flow chart showing the process of including and excluding articles

A critical narrative literature review was applied in this article. This was the preferred method in favour of the systematic review for its ability to cover current and a wide a comprehensive range of issues within a given topic [30, 31]. However, some features of systematic review methodology were used to reduce selection bias [32] by applying the Preferred Reporting Items for Systematic reviews and Meta-Analyses, which follows a protocol with clear methods that demonstrate [34] how many publications were identified and screened for eligibility, how many publications were excluded and why. The aim of the review was to identify literature on theoretical issues and evidence around the attainment of health equity of African minority within the New Zealand health delivery system. See Fig. 1. The electronic search included four databases, CINAHL, PubMed, Scopus, and Google Scholar published from 2010 onwards. Due to the drastic change in nature and growth of ethnic minority communities over the last two decades years, the authors believed that literature published before 2010 may have not provide the true picture of the current trend of health equity of African minority within the New Zealand health delivery system. Search terms used to select relevant articles are presented in Table 2.

**Table 2:** Search terms used to identify articles on health delivery system on African migrants

Search terms	
1. ‘Ethnic minority health’ and ‘New Zealand’	
2. ‘Ethnic minority’ and ‘healthcare’ and ‘New Zealand’	
3. ‘Ethnic minority’ and ‘healthcare’	
4. ‘Immigrants’ and ‘health’ and ‘wellbeing’	
5. ‘Migrants’ and ‘health’ and ‘wellbeing’	
6. ‘Immigrants’ and ‘health and wellbeing’	
7. ‘African migrant health’ and ‘wellbeing’ and ‘New Zealand’	
8. ‘African immigrants’ or ‘migrants’ and ‘healthcare’ or ‘health services’	
9. ‘Migrant’ and ‘healthcare’	
10. ‘Migrants’ and ‘health system’ and ‘New Zealand’	
11. ‘African migrant’ and ‘health’ and ‘wellbeing’	
12. ‘African’ and ‘immigrant health’ and ‘wellbeing’ and ‘New Zealand’	

The above key words were combined with derivations of the terms `equity’ OR `inequity’ (i.e. equity/equitable/equitably/equities/equitability, with the same derivations for inequity). Papers were excluded if they did not meet the following criteria: (i) studies that included New Zealand; (ii) have a focus on equity that influenced ethnic minority health outcomes in the delivering of healthcare; and (iii) articles for which full text was not available. Key details of the papers were summarised to include the name of the study and author, year title, study design and main result in respect to the building blocks. See Table 3.

**Table 3:** Studies by author, year title, study design and main result

Author(s), Year and title	Objective	Study design and sample	Main results (Presented into key services linking the six building block)
Huddleston, T., Bilgili, O., Joki, A., & Vankova, Z. (2015). Migrant Integration Policy Index 2015. http://www.mipex.eu/new-zealand	To assess, compare and improve integration policy	Literature review of national data on eight policy areas: labour market mobility, family reunion, education, political participation, long-term residence, access to nationality, anti-discrimination and health. Qualitative	1. Health services are well-prepared and responsive to the needs of diverse set of patients. Diagnostic and treatment methods are adapted to respond to specific cultural needs though mostly focused on Pacific Peoples and Māori. Cultural case workers are only available at a few specialised organisations. Free interpretation services are provided in dozens of languages through various methods.
Ministry of Business Innovation and Employment. (2019). Building and keeping a health workforce. Wellington: New Zealand Government. Retrieved from https://www.immigration.govt.nz/about-us/media-centre/newsletters/settlement-actionz/actionz3/building-and-keeping-a-health-workforce	To explore migration and health work force issues in the Western Pasific Region	Not mentioned	2. The building and keeping of a health workforce in New Zealand is in line with filling gaps of an elderly population.
Fouche, C., Henrickson, M., Poindexter, C. C., Scott, K., Brown, D. B., & Horsford, C. (2011). 'Standing in the Fire’: Experiences of HIV positive Black African migrants in New Zealand. Auckland, New Zealand. Retrieved from http://hdl.handle.net/2292/7362	To explore the lived experiences and social service needs of affected or at-risk Black African migrants, refugees and their family members in New Zealand	In-depth interviews with 13 HIV-positive Black African individuals Qualitative	1 HIV-related stigma affects participants’ access to care for HIV treatment. 3. Lack of HIV-related education provided to health professionals who live and work with this population outside the offices of the HIV specialty providers.
Came, H. (2012). Institutional Racism and the Dynamics of Privilege in Public Health (Doctoral thesis). University of Waikato, Hamilton, New Zealand. Retrieved from https://hdl.handle.net/10289/6397	To examine the extent and how is institutional racism and Pākehā privilege manifested within public health policy and funding practices.	Counter storytelling of 10 participants, a desktop review of Crown documents, an historical analysis, co-funding field notes, literature review, a survey of public health providers and a quantitative funding analysis. A mixed method approach.	There is institutional racism and structural inequality that determines: 1. Access and care processes for ethnic minorities; 2. Contractual practises of non-European workforce; 4. Funding allocation.
Auckland District Health Board. (2010). Youth Health Improvement Plan Youth Health Improvement Plan 2010-2014. Enhancing health and well-being for young people in Auckland District Health Board. Wellington. Retrieved from http://www.adhb.health.nz/assets/Documents/About-Us/Planning-documents/Youth-Health-Plan-2010.pdf	To improve the health status of young people living in Auckland city.	Literature review; Interviews (youth focus groups and stakeholders). Qualitative	1. Young people do not have equal access to services; 2. Workforce development is a key issue. There is a knowledge deficit amongst workforce trained to work with young people; 3. Information needs to be shared and coordination improved through a case management approach; 4. Projects lack funding for a coordination component.
Dumont, J., & Lafortune, G. (2017). Health Employment and Economic Growth: An Evidence Base. In J. Buchan, I. S. Dhillon, & J. Campbell (Eds.), International migration of doctors and nurses to OECD countries: recent trends and policy implications. Retrieved from https://www.who.int/hrh/resources/WHO-HLC-Report_web.pdf?ua=1	To examine trends in the international migration of health workers to Organisation for Economic Co-operation and Development (OECD) countries since 2000.	Examination of recent trends in the international migration of health workers to OECD countries since 2000 against the background of changes in migration and health policies, as well as changing economic and institutional circumstances. Literature review.	1. Health professionals are primarily from OECD countries like intra-European Economic Area, trans-Tasman and North American. In the African Region, immigrant doctors in OECD countries came primarily from Nigeria and South Africa and were mostly expatriates.
Dumont, J., & Zurn, P. (2007). Immigrant Health Workers in OECD Countries in the Broader Context of Highly Skilled Migration. In International Migration Outlook (pp. 161-229). Retrieved from https://www.oecd.org/migration/mig/41515701.pdf	To present a comprehensive and relevant picture of immigrants in the health sector in OECD countries, in order to better inform the policy dialogue at national and international levels.	Analysis of descriptive statistics provided on migrant health workers from origin and destination country to address lack of evidence on international migration of highly skilled health workers to OECD countries. Literature review	2. Recognition of foreign qualifications remains an important tool to insure high standards and quality in healthcare delivery, but also serves sometimes to control inflows of foreign-trained workers in OECD countries.
Health Central. (2018). Overseas doctors frustrated they can’t relieve GP shortage. Retrieved from https://healthcentral.nz/overseas-doctors-frustrated-they-cant-relieve-gp-shortage/	To explore the hurdle to being able to practice in New Zealand	Consultation with overseas-trained doctors and Medical Council. Analysis of Medical Council’s statistics. Qualitative	2. New Zealand has an acute shortage of medical doctors but resist to change policies that enable overseas medically doctors who reside in New Zealand to practice
Kanengoni, B., Andajani-Sutjahjo, S., & Holroyd, E. (2018). Setting the stage: reviewing current knowledge on the health of New Zealand immigrants—an integrative review. PeerJ, 6(e5184). doi:https://doi.org/10.7717/peerj.5184	To examine immigrant health in New Zealand	Integrative review of 28 articles on peer reviewed research articles on immigrant health in NZ.	2. Provision of health services to ethnic minorities by migrant health professionals with awareness, empathy and positive attitudes improve accessibility to health services.
Minister of Health. (2018). Vote Health. The appropriation estimates of 2018/19 (Vol. 6): Ministry of Health. http://planetmaori.com/Files/Content/2019/est18-v6-health.pdf	Not mentioned	Not mentioned	4. One off funding of NZD48, 000.00 was done by previous government on stock take of New Zealand health system for migrant in the year 2017/18. No budget was set for 2018/19 to 2021/22.
International Migration Outlook. (2015). Changing patterns in the international migration of doctors and nurses to OECD countries. In. International Migration Outlook, Retrieved from https://www.oecd-ilibrary.org/docserver/migr_outlook-2015-6-en.pdf?expires=1554798690&id=id&accname=ocid41012844&checksum=BD8BA8B9B6026AF4F00ED0C84B01DC0C. 10.1787/migr_outlook-2015-6-en.	To examine how the international migration of health workers to OECD countries has evolved since 2000	Analysis of data on the trend of international migration of health workers to OECD countries since 2000 from 120 countries. Literature review	2. Low recruitment of health workers from developing countries by OECD countries.
Ministry of Health. (2016). Health of the Health Workforce. Retrieved from https://www.health.govt.nz/system/files/documents/publications/health-of-health-workforce-2015-feb16_0.pdf	To synthesise information about the workforce and the environment it operates in, and from there to identify trends.	Review of multiple sources, including regulatory bodies such as: the Medical Council of New Zealand (MCNZ) and the Nursing Council of New Zealand (NCNZ); the wider Ministry of Health; DHBs and other employers; OECD1 reports; and New Zealand Census data. Literature review	2. There are calls to strengthen the health and disability workforce by improving the recruitment, retention and distribution of health professionals. Another key objective is to strengthen the health workforce intelligence and data needed to provide high-quality support and advice on current and projected gaps in the health workforce.
Ministry of Health. (2017). Annual Data Explorer 2016/17: New Zealand Health Survey. Retrieved from https://minhealthnz.shinyapps.io/nz-health-survey-2016-17-annual-data-explorer/_w_32a5d991/#!/home	To provide a snapshot of the health of New Zealanders through the publication of key indicators on health behaviours, health status and access to health care for both adults and children.	An interactive tool for exploring New Zealand Health Survey data on eight key indicators: psychological distress; self-related health; unmet need for a GP due to cost; unfilled prescription due to cost; obesity; current smoking; past year drinking and hazardous drinking. Data is represented by sex, age, ethnic group and neighbourhood deprivation.	2. There were no publicly available or accessible data or statistics on the health determinants, health system performance and health of African migrants. Results show data for the Māori, Pacifica and Asian populations.
Ministry of Health. (2014). Annual Update of Key Results 2013/14: New Zealand Health Survey. Wellington: Ministry of Health.	To identify key issues and monitor trends of the health behaviours, health status and access to healthcare.	80% of adults (13,309 adults) and 85% of parents/caregivers (representing 4699 children). Quantitative (survey)	1. 28% of adults and 22% of children had one or more types of unmet need for primary health care in the past 12 months. Unmet need takes various forms, including a person being unable to get an appointment at their usual medical centre within 24 hours and a person not going to a GP and/or after-hours medical centre when they had a medical problem due to cost or lack of transport. Neighbourhood deprivation is strongly linked to unmet need for primary health care. About one in three adults living in the most deprived areas (35%) had an unmet need for primary health care, compared with one in five (20%) of those living in the least deprived areas. 3. Although adults and children living in the most deprived areas report similar use of GPs over the last year to those living in the least deprived areas, they have much higher levels of unmet need for health care with cost being the main barrier. Adults and children living in the most deprived areas are more than three times as likely as those living in the least deprived areas to have not filled a prescription due to cost in the past year. These types of unmet need for health care are of particular concern where they affect people who are already in poor health.
Gray, Hilder, & Stubbe, 2012. How to use interpreters in general practice: the development of a New Zealand toolkit. Journal of Primary Health Care 4(1): 52-61	To identify the actual pattern of use of interpreters for migrants and refugees	Literature review on New Zealand’s District Health Boards’ policies on interpretation services	1. There is gross underuse of interpreters in the healthcare delivery system during access and care processes.
Mortensen, A. (2011). MELAA Report Summary. Nursing Praxis in New Zealand, 27(1).		Not mentioned.	MELAA groups face significant barriers to accessing health care including: 1. language and communication difficulties; health illiteracy in some groups; 2. A lack of cultural understanding by health service providers; and poor understanding of the New Zealand health system. 3. High cost of health care
Mortensen, A. (2011). Public health system responsiveness to refugee groups in New Zealand: Activation from the bottom up. Social Policy Journal of New Zealand (37), 1-12.	To examine the role of public health system in the integration of refugees.	A qualitative research using 28 in-depth semi-structured interviews with service providers in community, primary and secondary health care sectors, in both governmental and non-governmental agencies.	4. Specific health care settings for refugees and migrants are often poorly resourced.
Perumal, L. (2011). Health Needs Assessments for Middle East, Latin America and African people living in Auckland Region. Auckland: Auckland District Health Board. https://countiesmanukau.health.nz/assets/About-CMH/Performance-and-planning/health-status/2011-health-assessment-middle-east-latin-american-african-people-living-in-auckland.pdf	To investigate the population health trend of the Middle Eastern, Latin America and African (MELAA) in New Zealand.	Data analysis of the health and wellbeing of the MELAA group. Quantitative study	1. There are access issues within the health delivery system to include inadequate oral health care, poor health education and promotion on sexual health, family planning and antenatal care, and late and poor engagement with secondary mental health services for Africans; 2. There is a paucity of health workers from African ethnic minority groups within the New Zealand Health system. Providers of African ethnic profiling have perspective taking and empathy that contributed to improved health; 3. The struggle to meet health information needs, in terms of both the quality of data collected and the speed and clarity to which this information is made available is often related to grouping Africans with Middle East and Latin Americans into a single category, commonly known as MELAA or under the ‘other’ category label.
Russell, E. (2018). South African GP with over 14 years' experience waits a year and a half to sit Kiwi medical exams. New Zealand Herald. Retrieved from https://www.nzherald.co.nz/nz/news/article.cfm?c_id=1&objectid=12040593	Not mentioned	Interviewers. Qualitative study	2. Recognition of foreign qualifications remains an important tool to ensure high standards and quality in healthcare delivery. Overseas doctors wait one-and-a-half years to sit for New Zealand medical exams. The second of two exams only run three times a year and each sitting only has 28 spots available. When all exams are completed, overseas doctors wait five years for an internship and by that time their New Zealand medical certificate has expired.
Thomas, R. (2018). Foreign doctors struggling to get jobs in New Zealand. Stuff. Retrieved from https://www.stuff.co.nz/national/health/103932802/foreign-doctors-struggling-to-get-jobs-in-new-zealand	Not mentioned	Consultation with foreign doctors struggling to get jobs in New Zealand.	2. New Zealand has a “national immigration obligation” to prioritise New Zealand and Australian medical graduates for first year positions over foreign trained doctors.
Waitemata and Auckland District Health Boards. (2017). Asian, Migrant & Refugee Health Plan 2017-2019. Retrieved from http://www.waitematadhb.govt.nz/assets/Documents/health-plans/2017-19-Asian-Migrant-Refugee-Health-Plan-ADHB-WDHB-CPHAC-Final.pdf	Not mentioned.	Not mentioned.	3. Categorisation of Middle Eastern, Latin American and African in one category or as ‘other’ is problematic to inform, plan, and monitor services that target the unique needs of the ethnic groups separately. 5. Migrant health services are a priority in the Auckland Regional Settlement Strategy (Migrant and Refugee Health Action Plan) but not yet at national level.
Chin, M. H., King, P. T., Jones, R. G., Jones, B., Ameratunga, S. N., Muramatsu, N., & Derrett, S. (2018). Lessons for achieving health equity comparing Aotearoa/New Zealand and the United States. Health Policy, 122(8), 837-853. doi:https://doi.org/10.1016/j.healthpol.2018.05.001	To compare New Zealand and United State of America’s approaches to health equity to inform policy efforts	A comparison of Aotearoa/NZ and U.S approaches to health equity to inform policy efforts. Narrative review of literature.	Implemented policies are frequently not explicit in how they address health inequities, and often do not align with evidence-based approaches known to improve equity: 1. There are barriers to access and high quality care in both countries to include low health literacy, and limited cultural competence of providers. 4. Out-of-pocket costs are significant barriers to access to care. Co-payments to general practitioners (GPs) in Aotearoa/NZ are unaffordable to some and the uninsured in the U.S. often rely on charity care.
Gooder, C. (2017). Immigration, ethnic diversity and cities: A literature review for Auckland Council. Auckland: Auckland Council. http://knowledgeauckland.org.nz/assets/publications/TR2017-008-Immigration-ethnic-diversity-and-cities-literature-review.pdf	To investigate the social impacts of immigration-driven ethnic diversity and cities	A literature review on international and national literature on the social impacts of immigration driven ethnic diversity and cities.	1. People from visible minority ethnic categories are being racialized by the dominant New Zealand European culture where whiteness are normalised in institutions and systems such as health which is often linked to poor health outcomes.
Schneider, E. C., Sarnak, D. O., Squires, D., Shah, A., & Doty, M. M. (2017). Mirror, Mirror 2017: International Comparison Reflects Flaws and Opportunities for Better U.S. Health Care. https://www.commonwealthfund.org/sites/default/files/documents/___media_files_publications_fund_report_2017_jul_schneider_mirror_mirror_2017.pdf	To compare health care system performance in Australia, Canada, France, Germany, the Netherlands, New Zealand, Norway, Sweden, Switzerland, the United Kingdom, and the United States.	Seventy-two indicators were selected in five domains: Care Process, Access, Administrative Efficiency, Equity, and Health Care Outcomes. Data sources included Commonwealth Fund international surveys of patients and physicians and selected measures from OECD, WHO, and the European Observatory on Health Systems and Policies. Literature review	1. New Zealand performs well on measures of care process (prevention, safe care, coordination, and patient engagement) and administrative efficiency, but below the 11-country average on other indicators like accessibility, equity and healthcare outcomes.
Ward, C., Lescelius, J., Jack, A., Naidu, R. M., & Weinberg, E. (2018). Meeting the needs and challenges of migrants and former refugees in the Nelson and Tasman regions. Wellington: The Centre for Applied Cross-cultural Research, Victoria University of Wellington. https://www.victoria.ac.nz/__data/assets/pdf_file/0011/1470872/NMC-needs-analysis-final-reportMay2018.pdf	To explore health needs analysis for migrants and former refugees in the Nelson and Tasman region.	Qualitative research. 120 (46 males and 74 females, aged 14-79 years) migrants and former refugees from Asia (43%) and Europe (20%), followed by South and Central America (14%) and the Pacific (11%) with smaller numbers from Africa, the Middle East, North America and Australia. Participants had resided in New Zealand from less than a year to 43 years with the overall average length of residence being 7.5 years. 27% of the participants were students, 60% were employed, and 21% of the participants self-identified as being from a refugee background.	1. Language and communication barriers was the identified theme. The theme intersected with the remaining five themes: Systems and Services (inadequate information, poor services and few interpreters); Culture and Identity (limited New Zealand cultural competencies, maintenance of traditional culture, and intergenerational cultural gaps); and Health and Well-being (access to medical care and health risks).
Came, H. (2014). Sites of institutional racism in public health policy making in New Zealand. Social Science & Medicine, 106, 214-220.	To examine how institutional racism manifests in public health policy making and funding practice.	Mixed methodology: Document review of Ministry of Health policy documents from 1999 to 2011; a semi-structured interview, conducted with an upper echelon Crown official, to confirm operational practice.	1. Five specific sites of institutional racism were identified: majoritarian decision making, the misuse of evidence, deficiencies in both cultural competencies and consultation processes, and the impact of Crown filters. These findings suggest the failure of quality assurance systems, existing anti-racism initiatives and health sector leadership to detect and eliminate racism in health delivery system.
Harris, R., Cormack, D., Tobias, M., Yeh, L.-C., Talamaivao, N., Minster, J., & Timutimu, R. (2012). The pervasive effects of racism: Experiences of racial discrimination in New Zealand over time and associations with multiple health domains. Social Science & Medicine, 74(3), 408-415. doi:https://doi.org/10.1016/j.socscimed.2011.11.00	To investigate whether reported experience of racial discrimination in health care and in other domains was associated with cancer screening and negative health care experiences	The study uses data from the 2002/03 (n = 12,500) and 2006/07 (n = 12,488) New Zealand health surveys nationally representative population-based surveys of adults (15+ years).	1. New Zealand has shown reported experience of racial discrimination by a health professional to be higher among non-European ethnic groups with experiences of racial discrimination in different settings associated with multiple health outcomes and risk factors. There is a significant correlation between racial discrimination by healthcare providers with lower odds of preventive care.

1 = Service delivery; 2 = Health workforce; 3 = Health information system; 4 = Health financing system; 5 = Leadership and governance.

After an initial reading, the first author [BK] grouped the articles into five areas of the six WHO criteria for the six building blocks of health system strengthening concepts: 1) service delivery; 2) health workforce; 3) health information system; 4) health financing system; and 6) leadership and governance. Only one paper briefly mentioned the 5th criteria: equitable access to essential medical products, vaccines and technologies, which also fell under the theme service delivery. All authors agreed on these grouping themes.

## Results

The search retrieved 390 articles. After applying the criteria, 11 journal articles were included in the final analysis. Similar studies [34–36] with limited and or complex evidence, showed prescribed procedural search strategies to have failed to identify important literature. We therefore used informal approaches to retrieve grey literature using the same terms listed earlier, including international and New Zealand government websites and web archives known to specialise in ethnic minority health and which may provide important detail on the health system strengthening. The process also involved manually selecting relevant publications that were cited in the articles retrieved during the first search. The search found an additional 7 government documents on organisational websites: Auckland and Waitemata District Health Boards; Ministry of Business, Innovation and Development; and Ministry of Health and four from international websites. An additional included grey literature from media (three), and three from reference tracking brought the total number of articles included in the review to 27. Eight were qualitative studies, four were quantitative studies, two used a mixed methodology strategy, and nine studies were literature reviews. Four studies did not mention the methodology used. Of these 27, only nine explicitly mentioned the African community in New Zealand.

## Findings

### Service delivery: access and processes of migrant health services

18 peer-reviewed articles [13, 14, 25, 37–46], one inter-government publication [9], one Ministry of health document [47], and one report from Auckland District Health Board [27] were found to focus on access and processes suggesting its importance in health care delivery analysis. According to the 2015 Migrant Integration Policy Index, the New Zealand’s legislation confirm a range of rights regardless of legal status, including access to free basic healthcare [9]. However, there is contradictory evidence showing these rights not to be fulfilled, protected and promoted [37–42]. For instance, in their measuring of health care system performance of 11 countries in the Organisation for Economic Co-operation and Development (OECD) with respect to care process, access, administrative efficiency, equity, and health care outcomes, Schneider et al., [39] ranked New Zealand in the eighth place, with a score of − 0.24. The performance score was based on the distance from the average of the 11 countries. Performance issues included: accessibility (language barriers and denial of access based on lack of documentation; negative healthcare provider attitudes and delays in the health system); equity; and health outcomes.

Some findings argue the existing access and process of migrant health services is poor due to lack of information and communication. They report practical access to information and the provision of interpreters to be lacking [44, 45], which in turn are a requirement of ethnic minorities to address their informational needs to facilitate access to and understand the operation of basic medical systems and services including a visit to the general practitioner, obtaining medicines, and gaining access to specialist care [37, 38]. The studies also acknowledged the limited cultural competence of providers and out-of-pocket costs (high co-payments to general practitioners), which were often unaffordable to the ensuing migrant communities.

There is also evidence to show a significant correlation between racial discrimination by healthcare providers with lower odds of preventive healthcare for ethnic minorities [13, 37, 42, 43]. Although majority of the studies specifically refer to the indigenous Māori ethnic group, these problems equally affect the African community, as evidence has reported racial discrimination by a health professional to be higher among non-European ethnic groups in different settings associated with multiple health outcomes and risk factors [25, 41, 48]. For example, one study showed Africans to have presented a higher cost of dispensed pharmaceuticals per person on Human immunodeficiency virus/Acquired Immune Deficiency Syndrome (HIV/AIDS) related healthcare but a lower value of nominal costs per person for laboratory tests compared with others [25]. On the contrary, one study reported HIV/AIDS participants of African descent to receive a higher quality of HIV related healthcare in New Zealand than in Africa [46]. However, the authors noted the comments appeared to be related to fears of being deported and consequently losing the available care.

Findings also showed a mono-cultural practice, displayed as institutional racism, which systematises healthcare into one dominant cultural model in favour of the New Zealand European population to deter access and care processes. Gooder [40] argued with the support of findings from other studies [39, 41, 42] that people from visible minority ethnic categories, like black Africans, are being racialized by the dominant New Zealand European culture. She described dominance not from a numerical perspective, but rather as cultural dominance illustrated by health system. This is in tandem with other studies which revealed concerns for Africans around inadequacy and or culturally inappropriate health education and promotion on sexual health, family planning and antenatal care, and mental health services [25, 45]. The studies, however, overlooked the examination of the overall health systems context or requirements for a more efficient network of services.

### Limited African health workforce to provide healthcare to their communities

Findings show a paucity of health workers from African ethnic minority groups or those with a background understanding of the African community within the New Zealand health system [25, 49]. Although having a workforce from the African community does not automatically transfer to guaranteed health equity for Africans, some studies have recommended the delivery of healthcare, health promotion and education, health campaign by African health professionals and or those who understand the norms and cultural etiquette when working with African migrants as key to improve health outcomes amongst Africans [25, 27, 45, 46, 49, 50]. Evidence shows New Zealand to source healthcare workers from the major English-speaking background OECD countries for the purposes of filling gaps in its ageing health workforce [49, 51, 52]. Low recruitment of health workers from Africa are attributed to codes of conduct that hinder targeted recruitment from developing countries who are experiencing shortages of healthcare staff [53, 54]. However, many African health care professionals who migrate on their own still fail to practise in New Zealand [40–42]. Rigorous assessment of foreign healthcare qualifications remains an important mechanism to ensure high standards and quality in healthcare delivery [40–42], but appears to serve largely to control inflows of foreign-trained workers [51, 53]. In addition, a number of studies also showed evidence in the differential treatment of ethnic minorities, across different providers whereby non-European New Zealand health providers were given shorter contracts and were more often than not under scrutiny [14, 41, 43].

### Health information systems

There were no publicly available or accessible data or statistics on the health determinants, health system performance and health of African migrants within the Ministry of Health databases [47, 55], suggesting the health information system to have failed to produce, analyse, disseminate and the use of timely information on this group. On the contrary, the databases show references and published health reports on Māori and Pacific population dating back to the 1990s were available. Similarly, health information reports on Asian migrants are better off than those for Middle East, Latin Americans and Africans (MELAA) groups [27, 56]. Findings show the struggle to meet health information needs, in terms of both the quality of data collected and the speed and clarity to which this information is made available is often related to the current New Zealand Demographic and Household Survey. This survey, continues to group Africans with Middle East and Latin Americans into a single category, commonly known as MELAA or under the ‘other’ category label, which group widely different cultures together in a perceived homogeneity with a common denominator of being poor, underdeveloped, and helpless. The same phenomenon has been found for Asian diverse ethnic groups [56].

### Leadership and governance

Responsiveness at local level is not any better. Good governance is not possible without a strong health information system. The scarcity of research on how health policies and reforms have affected health equity for ethnic minorities, is not surprising. Suffice to say, migrant health is a priority in the Auckland Regional Settlement Strategy (Migrant and Refugee Health Action Plan) but not yet at national level [56]. Findings show the national policy environment to be giving unclear inconclusive direction on migrant health, with tensions between national and local strategic goals.

### Health financing

As noted in Table 1, health financing aims to raise adequate funds for health, in ways that ensure people can use needed services, and are protected from financial catastrophe or impoverishment associated with having to pay for them. Vote Health is the primary source of funding for New Zealand’s health and disability system [57]. In 2018/19 financial year, findings show $13,236 million (72.6% of the Vote) was provided to the 20 district health boards (DHBs) for services to meet the needs of each district’s population, taking into account regional considerations, government priorities, and the strategic direction set for the health sector. Generally, specific health care settings for ethnic minorities are often poorly resourced [57]. Not only did findings show a one off funding of NZD48, 000.00 on stock take of New Zealand health system for migrant in the year 2017/18, no budget was set for 2018/19 to 2021/22, with exception for workforce allocation and the strategies for Asians [56]. As mentioned in the health financing system building block, the construct of an African ethnic grouping in New Zealand provides a banner or label under which the needs of this community are not recognised or visible enough to be worthy to finance its health issues. This includes financial inaccessibility of interpretation services which were found to be mostly located in specialised organisations and departments [9, 44]. Resultantly, no findings were found to show a clear dedicated budget for Africans. However, it is worthy to note financing for health services is not only an issue for the African migrant population in New Zealand, but for Middle East and Latin America with some exceptions for the Asian community [45, 56]. Accordingly, areas where Africans predominantly live in Auckland, Avondale-Roskill ward, is the most medically deprived in the city with disproportionately high rate of hospital admissions [27, 47].

## Discussion

The review shows a severe dearth of studies specifically related to the African ethnic minority population’s health needs and challenges residing in New Zealand, within the WHO concept of health systems strengthening [3]; which incorporates six inter-related health system building blocks: 1) service delivery; 2) health workforce; 3) information; 4) medical products/vaccines/technologies; 5) financing; and 6) leadership and governance. In general, huge disparities exists across different building blocks of health systems addressing the health needs and rights of ethnic minorities, including those in African communities.

Historically, New Zealand health policies and services for ethnic minorities, has been more responsive to the needs of indigenous Māori ethnic group, than other ethnic minorities who came as migrant and refugees New Zealand. Therefore, the accounts offered in this review in regards to the building block – service delivery, describe governmental policies and programmes not to address factors hindering access to healthcare of ethnic minorities as they do with the Pacific and Asian community [17, 56]. Acknowledged is the lack of culturally sensitive and responsive health service delivery, which unfortunately manifests as a top-down and ‘one size fits all’ service. Consequently, the healthcare delivery system has not been culturally adapted and made sensitive for Africans alongside populations from Middle East and Latin American grouped as MELAA categorisation which in itself is problematic when used to inform, plan, and monitor services [56] as many health differences are masked. This promotes the invisibility of African health inequity within all the other building blocks especially the health information system and health financing system.

Accordingly, the New Zealand health information system has not been able to adequately capture the ethnic dimension of African communities. This may be attributed to the continuous use of an inaccurate description of an African migrant without any critical reflection on the inherent discrepancies it creates by underestimating the actual African population in New Zealand. Callister [58] notes the process and politics of ethnic enumeration to inform New Zealand policy formulation and resource allocation. Inaccurate numerical presentation of African migrant communities and subsequently inaccurate representation of their needs may explain to some extent the underfunding or lack of funding of migrant health services like interpretation services. It is therefore not surprising New Zealand amongst other countries which has migration inflows, has a publicly funded healthcare system which is correlated with out-of-pocket private health spending and not with public health expenditure. Further, targeted District Health Board funding outside of mainstream funding is limited due to the equity focus on Māori and Pacific health outcomes at present.

For a multi-cultural society like New Zealand, ones would assume that a major feature of the country’s health workforce would be a diversified workforce. Poynter et al., [59] viewed it as an implicit bias in the way services are set up, where a health workforce from English-speaking countries are prefered, yet they are the least likely to stay, compared to overseas trained doctors from Africa, Middle East and Asia [49]. Although having health professionals of African backgrounds does not guarantee improvement of health services for African communities, yet when such professionals are available, awarding shorter contracts, or lack of African trained health professionals may be hindrance to the effectiveness healthcare service delivery to the African community. At the same time, the lack of African cultural understanding and African culturally appropriate education by the health service providers, have been noted to affect the confidence of African communities to approach services [25, 50]. African migrants have shown preferences for their own cultural context of care in their hospital encounters, an example of which includes traditional confinement practises for the mothers post natally [60], which are often contested within the western healthcare model [37].

Leadership and the governance of the New Zealand health systems for improving health outcomes for the poor often coincide with ethnic minority status. At the national level, uncertainty and incongruity on the inclusion of migrant health in the public health system, continue to be pose challenges to health equity. For example, Auckland (the largest city in New Zealand) has the highest number of immigrants, with 40% of its population being born overseas and almost half of the total African population, live, study and work in Auckland [6]. Ironically, the identified health needs of the migrants within the Auckland region were not catered for in the Auckland Regional Strategic Plan 2005–2010 as they were not recognised as a priority group in national health policy or in reducing inequalities. Each District Health Board use discretion on what and how they allocate the population-based funding to their populations based on the equity needs of the Māori and Pacific community.

## Recommendations and conclusion

The New Zealand health delivery system continues to perform reasonably well, but inequities remain high for visible ethnic minority migrants like Africans who have limited or no access to healthcare. There is a need to strengthen the health system derived from a well-informed funding allocation relevant for health services needed by the African communities. Furthermore, there should be strategic discussions followed by action to address structural and institutional racism and white privilege in order to explicitly address root causes of inadequate access and care processes for ethnic minorities.

The health information system must be strengthened to include employing context relevant household surveys or other means to gather specific demographic and health baseline data from African migrant communities. This would need to include a categorisation label for Africans in the health information system to allow for monitoring and timely dissemination of information. Such an initiative would in turn then assist in resource allocation and adjusting of some building blocks to meet the needs of this population. The New Zealand government should also start conducting time series reports that provide trends in health outcomes and utilisation of health services by the African minority group to allow for a more valid and comprehensive picture of their health in New Zealand.

Workforce development is a key for ensuring improved health outcomes for Africans. It includes relevant training and the provision for a health workforce which works in ways that are responsive, fair and efficient to achieve the best health outcomes possible, given available resources and circumstances; that is; there are enough numbers and ethnic mix of staff, fairly distributed; they are competent, responsive and productive. African health professionals already residents of New Zealand should receive equal opportunities for practicing in keeping with other professions from different ethnic backgrounds. Such engaged professionals would also be much less likely to have a racial bias which would have major implications on how services are delivered and acceptance by the African healthcare user.

There is generally a need for more research and political will to strengthen the WHO building blocks within the current New Zealand health system service deliveries to be more responsive to the health needs and rights of African minority and other ethnic minorities. Whilst, this review acknowledges the lack of quantitative research, it appraises qualitative research to be best suited to answer the questions of ‘why’ and ‘how’. Such knowledge is needed to design interventions and take affirmative action. Nonetheless, the use of both qualitative and quantitative analysis is recommended to measure health equity within the health system for African minorities. Intrinsic to such research is a strong engagement with African minorities and the use of a multi-pronged data collection approach, and a reliance on an all-encompassing use of a cultural element to maximise the trustworthiness of findings.

### Study limitation

The results are presented as a narrative review due to a limited number of articles and data not well corresponding with the subject matter. However, to reduce such bias, the research strategy in the selection of articles adopted some aspects of systematic review methodology.

## Data Availability

All data generated or analysed during this study are included in this published article.
